# A theory of change for community interventions to prevent domestic violence against women and girls in Mumbai, India

**DOI:** 10.12688/wellcomeopenres.15128.2

**Published:** 2019-08-21

**Authors:** Nayreen Daruwalla, Surinder Jaswal, Prakash Fernandes, Preethi Pinto, Ketaki Hate, Gauri Ambavkar, Bhaskar Kakad, Lu Gram, David Osrin

**Affiliations:** 1 Program on Prevention of Violence Against Women and Children, SNEHA, Mumbai, Maharashtra, 400017, India; 2 School of Research Methodology, Centre for Health and Mental Health, School of Social Work, Tata Institute of Social Sciences, Mumbai, Maharashtra, 400088, India; 3 Independent Researcher, Mumbai, Maharashtra, 400050, India; 4 Institute for Global Health, University College London, London, WC1N IEH, UK

**Keywords:** Domestic violence, gender-based violence, intimate partner violence, theory of change, India, Mumbai

## Abstract

**Background:** We describe the development of a theory of change for community mobilisation activities to prevent violence against women and girls. These activities are part of a broader program in urban India that works toward primary, secondary, and tertiary prevention of violence and includes crisis response and counselling and medical, police, and legal assistance.

**Methods:** The theory of change was developed in five phases, via expert workshops, use of primary data, recurrent team meetings, adjustment at further meetings and workshops, and a review of published theories.

**Results:** The theory summarises inputs for primary and secondary prevention, consequent changes (positive and negative), and outcomes. It is fully adapted to the program context, was designed through an extended consultative process, emphasises secondary prevention as a pathway to primary prevention, and integrates community activism with referral and counselling interventions.

**Conclusions:** The theory specifies testable causal pathways to impact and will be evaluated in a controlled trial.

## Introduction

Although its pervasiveness and harms have long been addressed by the women’s movement, the importance of violence against women and girls as a public health priority has only been acknowledged relatively recently. The health effects are profound. Violence causes non-fatal or fatal injury: 21% of homicides in southeast Asia are committed by an intimate partner, constituting 60% of all female homicides (compared with 1% of male homicides) ( Stöckl *et al.*, 2013). Other harms include sexually transmitted infections, miscarriage, induced abortion, stillbirth, low birth weight, preterm delivery, harmful drug and alcohol use, anxiety and depression, self-harm, suicide, and trans-generational recapitulation of violence ( García-Moreno *et al.*, 2015b; WHO and London School of Hygiene and Tropical Medicine, 2010; WHO, 2013). Physical and psychological trauma and fear also lead to mental health problems, limited sexual and reproductive control, somatoform conditions ( WHO, 2013), difficulties in seeking healthcare, and lost economic productivity ( Solotaroff & Pande, 2014).

Some 30% of women have experienced physical or sexual violence by an intimate partner or sexual violence by a non-partner ( WHO, 2013). A recent systematic review suggested that 22% of women in India had survived physical abuse in the past year, 22% had suffered psychological abuse, 7% sexual abuse, and 30% multiple forms of violence ( Kalokhe *et al.*, 2017). Non-partner sexual violence is reported regularly in the media ( Raj & McDougal, 2014), but culturally sanctioned household maltreatment ( Silverman *et al.*, 2016) in the form of emotional and economic domestic violence and abuses of power, control, and neglect have been reported as particularly common in India ( Kalokhe *et al.*, 2015).

India was one of 189 signatories to the 1980 Convention on the Elimination of All Forms of Discrimination Against Women ( United Nations, 1979). The United Nations declared a response imperative in 2006 ( United Nations, 2006), the World Health Organization (WHO) named it a health priority in 2013 ( García-Moreno *et al.*, 2015a), and its elimination is a target of the fifth Sustainable Development Goal. The emphasis of the first wave of interventions—driven largely by feminist activism by the women’s movement from the 1960s—was support for survivors of violence, reduction in secondary perpetration, strengthening legal recourse, and advocacy ( Ellsberg *et al.*, 2014). This constitutional, rights-based approach led to the consolidation of services such as women’s shelters, counselling, legal advice, and, in India, laws such as the Protection of Women from Domestic Violence Act 2005. A second wave of interventions, again led by civil society organisations, emphasized primary prevention and community activism and took a public health position which emphasized population-based, interdisciplinary, and intersectoral interventions ( WHO and London School of Hygiene and Tropical Medicine, 2010).

The Society for Nutrition, Education and Health Action (SNEHA) is a non-government organisation addressing the health needs of women and children in the context of urban informal settlements in India. The program on prevention of violence against women and children follows a socio-ecologic model developed by Heise after the work of Bronfenbrenner ( Bronfenbrenner, 1979; Heise, 1998), with an understanding that determinants of violence need to be addressed at a range of levels, within families, communities, and societies. The program aims to develop strategies for primary prevention, ensure survivors’ access to protection and justice, empower women to claim their rights, mobilise communities around ‘zero tolerance’ for violence, and respond to the needs and rights of neglected groups.

The program delivers three sets of activities: community mobilisation, crisis counselling and extended response for survivors of violence, and work with police, medical and legal services. Community mobilisation includes group activities and individual voluntarism. Neighbourhood groups of women, men, and adolescents develop awareness, initiate campaigns and local action to support survivors, and build leadership. An emergent cadre of volunteers *sanginis* (female friends) identify and support survivors through crisis intervention and case management, linking them with counselling, police, and medical services. This encompasses both primary and secondary prevention. The program runs five community-based and four hospital-based counselling centres. Immediate and longer-term support for survivors of violence is provided by counsellors at each centre. Counsellors take a stepped-care approach to identification, intervention, and referral of survivors of violence with common and severe mental health disorders, including in-house psychologists when required. They also work with medical, legal, and police services, for whom collaborative training programs are regularly conducted. Counsellors collaborate with the police and District Legal Aid Services Authority to assist women in filing legal cases in response to domestic violence, sexual assault and rape, and other matters pertaining to civil and criminal acts.

Our program is comprehensive and aspires to ‘community building’. Its characteristics include horizontal complexity (across sectors), vertical complexity (across socio-ecologic levels), community building (participatory efforts to enhance the capacities of individuals and connections between them and outside resources), political, economic, and infrastructural contextual issues with little power to affect them, flexibility over time, and community saturation (clusters rather than individuals) ( Auspos & Kubisch, 2004).

After 15 years of cumulative program development, we wanted to understand how the components of the program fit together, think about the sequence of outcomes and indicators that we might measure, and evaluate effectiveness. We contemplated expansion and felt a need to crystallise the program for ourselves, for others, and for protocolised rollout. We felt that the service aspects of the program were intrinsic to a rights-based response in the spirit of the Istanbul Convention ( Council of Europe, 2011). Community mobilisation is less defined, despite its potential to prevent violence against women and girls ( Ellsberg *et al.*, 2014), and the theory of change focused on it for this reason. A theory of change is a hypothetical explanation of how and why an initiative works ( Weiss, 1995). It seeks to understand how program activities might lead to outcomes—desired or undesired—by articulating the connections between them ( Stein & Valters, 2012). Each program activity is linked with outcomes and each outcome is defined and assigned indicators ( Taplin *et al.*, 2013). Like a logic model, a theory of change is a kind of program theory ( Rogers *et al.*, 2000), or pragmatic framework ( De Silva *et al.*, 2014), in which concepts are linked with empirical findings in steps that are potentially examinable and falsifiable. Shaping evaluation around theories of change has been recommended for a variety of social programs ( Chen & Rossi, 1980; Chen, 1994). Evaluators in the field of health promotion were early adopters ( Birckmayer & Weiss, 2000), and there has been growing interest in evaluating complex public health interventions ( De Silva *et al.*, 2014). We were particularly interested in developing a theory of change for the prevention of violence against women that fit our specific context of work among informal settlements in urban India.

We developed a program theory of change informed by existing theories around social norms, networks, and behaviour change ( Davidoff *et al.*, 2015; Michie *et al.*, 2011), combined with tacit theory based on experience ( Birckmayer & Weiss, 2000; Chen & Rossi, 1980; Mason & Barnes, 2007; Weiss, 1997). Our focus was on two general types of behaviour: stimulation of pro-social action and bystander intervention, and identification, support, and secondary prevention for survivors of domestic violence. In this paper, we aim to describe our theory of change.

## Methods

### Setting

Informal settlements (slums) are features of urbanization in India and have been described in two-thirds of cities and towns. The most recent estimate is that 41% of Mumbai’s households are in such settlements ( Chandramouli, 2011). The latest National Family Health Survey (NFHS-4) suggests that 21% of ever-married women in Maharashtra state, the location of our work, have experienced intimate partner violence in their lifetime ( IIPS, 2015). Risk factors for both physical and sexual violence include poverty, exposure to parental violence, childhood maltreatment, limited education, unemployment, young adulthood, mental disorder, substance use, individual acceptance of violence, weak community and legal sanctions, and gender and social norms supportive of violence ( WHO and London School of Hygiene and Tropical Medicine, 2010). These risk factors meet in Mumbai’s urban informal settlements, along with population density and stressful living conditions, and their toll in terms of violence is the reason for our activities. UN-HABITAT characterizes them in terms of overcrowding, insubstantial housing, insufficient water and sanitation, lack of tenure, and hazardous location ( Ministry of Housing and Urban Poverty Alleviation, 2010; United Nations Human Settlements Program (UN-Habitat), 2003). Women and girls in these communities lack both financial and social resources and an understanding of the possibility of relief from endemic violence.

### Activities

We developed the theory of change in five overlapping phases. In the first phase (July 2015 to November 2016), an external consultant (Fernandes) met with our teams for counselling and community mobilisation, police and hospital liaison, two clinical psychologists, and a lawyer to understand their experiences, challenges, and perceptions of outcomes. He interviewed seven clients of our crisis and counselling services, six police officers, and five healthcare providers, and conducted focus group discussions with eight members of a community women’s group, 15 members of a men’s group, 17 members of a youth group, and nine adolescents involved in an education program (report available as extended data ( Osrin, 2019)).

To begin the second phase, we convened a three-day research workshop (August 2015) with nine team members and four researchers in the field of violence against women and girls, anthropology, ethics, and public health. The discussions were primarily about outcomes: whether the impact towards which the theory of change would be directed was a reduction in violence against women and girls, gender-based violence, intimate partner violence, domestic violence, or violence perpetrated by others outside the home. The decision, supported by subsequent discussions, was to focus on domestic violence against women and girls.

The third phase, bracketed by workshops at the beginning and end involving members of the core team, data collectors, and SNEHA researchers, interrogated the first draft of the theory of change in terms of program experience and ethics. Participants discussed and refined potential outcomes, how they were related to preventing violence, and how they could be measured. This was accompanied by two activities: an action documentation exercise and a study on gender norms around domestic violence ( Daruwalla *et al.*, 2017). In order to understand what kinds of individual and collective action people might take, and therefore to align our expectations in the theory of change with potential reality, the action documentation exercise recorded community mobilisation team members’ experiences of the kinds of action that community members had undertaken in the past. It yielded 76 actions, documented as extended data ( Osrin, 2019). Adverse effects were also documented (although flare-ups in communities might actually suggest that the program was having an effect).

Table 1 provides examples of actions, categorising them as individual, collective, or individual followed by collective, whether an action was in the home or the neighbourhood, and the type of violence to which it was a response. The core team selected stories purposively to illustrate combinations of these categories. Individual action was often backed by the notional and practical security of being a member of a community group and having connections with a supportive organisation, using these connections as action escalated. Apart from help from our own organisation, and from periodic referral for skilled legal help and services for alcohol and drug dependency, the police force was a prominent institutional link. Although our commitment is to preventing domestic violence, both individual and collective actions often responded to incidents in the neighbourhood. Different kinds of violence – and particularly sexual violence in public spaces – coexist and response to each is both a source of confidence and a step toward a belief in collective efficacy. Likewise, groups often undertook collective action to improve their environment and we see this as contributing to a sense of collective efficacy and community building.

**Table 1.** Illustrative examples of past actions described in a documentation exercise.

Incident and action	Individual or collective	Ecologic level	Form of violence	Intervention function *
A community organiser persuaded a survivor of violence to seek help in a situation in which her husband was a local crime lord.	Individual	Home	Physical	Education Persuasion Enablement
A *sangini* helped a bride-to-be deal with dowry demands from the groom’s family by explaining the law and her rights and then discussing them with the family.	Individual	Home	Economic	Education
A woman told a community organiser that her neighbour was being beaten and locked in the house by her partner. The community organiser organised campaigns in the area and gathered a group who visited the house repeatedly, heard the woman inside, and got the police to effect entry. The woman and her partner were counselled by our organisation and continue to live together.	Individual to Collective	Home	Physical	Education Persuasion Enablement
A *sangini* intervened when a couple were fighting in the street, accompanied by their two young children. They had doused themselves in kerosene and were threatening to set fire to themselves. Other residents appeared and the *sangini* called the police. The couple returned later to thank the *sangini*.	Individual to Collective	Home	Physical	Persuasion Coercion
A *sangini* heard her neighbour’s seven-year-old daughter shouting for help when a local man attempted to rape her. She gained access to the house, prevented him from leaving, and called for help. A group of neighbours took him to the police station, where he was arrested.	Individual to Collective	Home	Sexual	Coercion
A woman fled to her mother’s home after her partner hit her, but he came after her and attacked both her and her mother. They shouted for help and other group members detained the man and called the police, who arrested him. The couple are in a counselling program.	Collective	Home	Physical	Coercion Education
Counsellors convinced representatives of a community body that a woman who separated from her violent husband could accept alimony without shame	Collective	Home	Economic Physical	Education Persuasion
A young married woman was being harassed by two gang members. When she rejected their advances, they beat her up, set her on fire and locked her in her house. At the public mourning after her death, a large group of relatives and friends banded together and sought help from our organisation to form a group. Our organization communicated with both the group and the police to make sure that process was followed and the perpetrators were jailed.	Collective	Neighbourhood	Sexual Physical	Persuasion Coercion
A *sangini* led a women’s group to repeatedly confront a group of drug users who were harassing women. The harassment stopped and the *sangini* became a community leader in a male-dominated area.	Collective	Neighbourhood	Sexual	Persuasion Modelling
Groups worked together to clean up localities, so successfully that the municipality made an educational video about them.	Collective	Neighbourhood	Environmental	Modelling
Groups called public meetings and deputations to the municipal corporation in order to have the local water supply fixed.	Collective	Neighbourhood	Environmental	Modelling Enabling Persuasion

*Education, Persuasion, Enablement, Coercion, Incentivisation, Training, Modelling, Environmental restructuring, Restrictions ( Michie *et al*., 2011)

We also tried to classify behaviour change according to the Capability, Opportunity, and Motivation model (COM-B), a theoretical approach that we will use in subsequent process evaluation. Developed to inform public health programming, the COM-B model specifies nine functions by which an intervention might stimulate change: education, persuasion, incentivisation, coercion, training, restriction, environmental restructuring, modelling, and enablement. The examples in Table 1 point to education (awareness of the problem of violence and potential approaches to addressing it), persuasion (of either perpetrators or community bodies), coercion (primarily of perpetrators, and often through linkages with the police), and modelling (of exemplary successful actions by individuals and groups) as prominent functions.

The fourth phase involved collective adjustment of the theory of change. The program core team (six SNEHA program and research members and one UCL researcher) met 15 times to work on the theory, for 2-3 hours each time. During these meetings, we used ‘backward mapping’, in which we started with agreed outcomes and then stepped backwards sequentially to understand the necessary preconditions to meet them. This was accompanied by examination of assumptions and rationales, a strategic weighing of possible interventions, and the development of indicators with which to test the causal sequence ( Taplin & Clark, 2012). At the end of this phase, we presented, discussed, and adjusted the emerging theory in four workshops. We invited external activists, academics, and practitioners in Mumbai, our program base (April 2016), Delhi, where policy and advocacy expertise is concentrated (July 2016), and London, our collaborative academic base (July 2016). A summary of these meetings is available as extended data ( Osrin, 2019).

In order to make sure we had not missed anything, the fifth phase involved a more formal review of mechanisms in the literature (PROSPERO 2018 CRD42018093695 Available from: https://www.crd.york.ac.uk/prospero/display_record.php?ID=CRD42018093695; a full review is forthcoming). We carried out a search for theories of change, logic models, or conceptual models of population-based interventions to prevent domestic violence. We included articles in English and excluded studies from high-income settings according to World Bank classifications. We limited the search to articles published between January 1960 and November 2018. We used Boolean combinations of the terms ("theory of change" OR "logic model" OR "conceptual model") AND ("intimate partner violence" OR "domestic violence" OR "violence against women" OR "sexual violence" OR "physical violence" OR "economic violence" OR "emotional violence" OR "gender-based violence") to search for articles on PubMed, Scopus, Web of Science, Google Scholar and Google sites. To limit the scope of the search, we inspected only the first 10 pages of Google Scholar and Google searches. We also read published impact evaluations listed in existing evidence reviews of interventions seeking to reduce domestic violence ( Bourey *et al.*, 2015; Ellsberg *et al.*, 2014; Gibbs *et al.*, 2017; Marshall *et al.*, 2018; Yount *et al.*, 2017).

### Ethical approval

Approval for research associated with the development of the theory of change was given by the Ethicos Independent Ethics Committee (ref: 3 ^rd^ December 2015). Participants in interviews and focus groups provided written consent to use of anonymised information.

## Results

### Primary and secondary intervention

**Figure 1.**
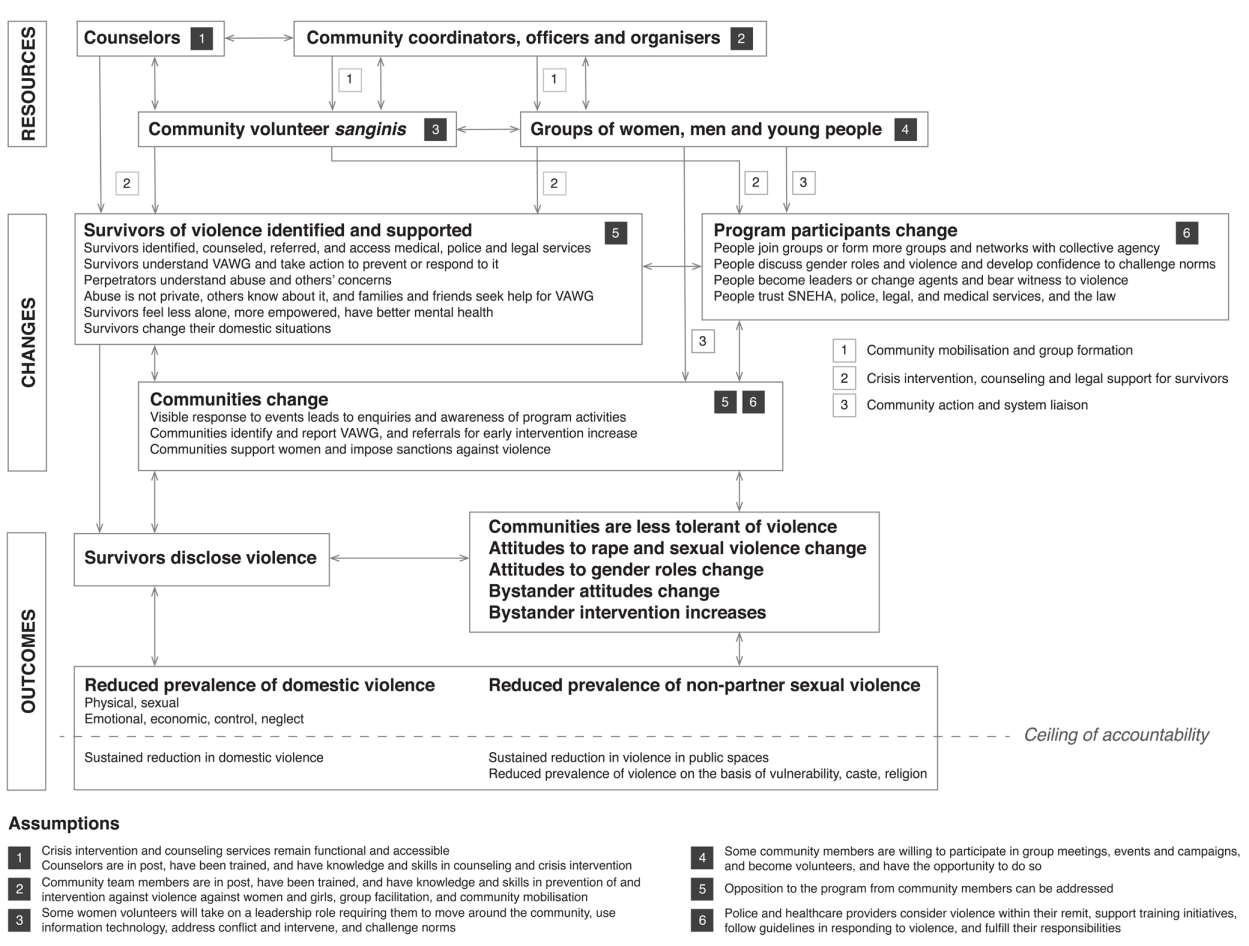
Theory of change.

Figure 1 summarises the emerging theory of change over a sequence of human resources or inputs, changes or outputs, and outcomes. The assumptions underlying the model are numbered and the activities of people involved are numbered and linked with a summary in Table 2. Raw data from interviews and focus groups are available ( Osrin, 2017).

**Table 2.** Community interventions to prevent violence against women and girls.

**1**	**Community mobilisation and group formation**	
	Community organisers	• Identify community members who want to join groups of women, men, and young people
		• Convene and facilitate groups
		• Share experiences and deliver modules on gender norms, understanding violence against women and girls, vulnerabilities to it and appropriate responses, rights, and negotiation skills
		• Identify women who want to become volunteer *sanginis*
**2**	**Crisis intervention, counselling and legal support for survivors**	
	Community volunteer *sanginis*	• Identify incidences of violence against women
		• Assess safety, provide initial counselling and information on rights and law to survivors
		• Record incidents, negotiate action, and intervene to ameliorate conflict
		• Arrange referral to SNEHA.
		• Organise temporary shelter and childcare
		• Support survivors in accessing family interventions and police or health services
		• Locate perpetrators of violence and negotiate with them and families
		• Conduct community follow-up
	Community organisers, officers, coordinators	• Support *sanginis* in their field activities
		• Provide support to survivors of violence and coordinate family interventions
		• Arrange referral to SNEHA, police, or health services
		• Coordinate and undertake community follow-up
		• Act as main contact between community and counselling teams, and communicate with stakeholder networks
	Groups of women, men, and young people	• Identify incidences of violence and inform *sanginis* and community officers
		• Intervene to ameliorate conflict
		• Arrange referral to SNEHA
		• Support survivors of violence in accessing family interventions and police or health services
		• Locate perpetrators of violence and negotiate with them and families
	Counsellors	• Provide crisis counselling and intervention services
		• Counsel survivors of violence and their families
		• Make home visits for crisis intervention, family discussions, and follow-up
		• Organise referral to police, health and legal services and negotiate with them
		• Assess survivor mental health and refer for therapy
**3**	**Community action and system liaison**	
	Community organisers, officers, coordinators	• Organise and participate in community campaigns and support visible collective action
		• Liaise with police and health providers and negotiate with community bodies
	Groups of women, men, and young people	• Participate in community campaigns and contribute to visible collective action
		• Liaise with police and health providers and negotiate with community bodies
		• Negotiate with municipal representatives for infrastructure and entitlements
	Community volunteer sanginis	• Support and participate in community campaigns and contribute to visible collective action
		• Liaise with police and health providers and negotiate with community bodies

The theory of change narrative is that people involved in our intervention take action to help survivors of violence make informed choices, and to increase community awareness of violence against women and girls and the possibility of change. As a result of these activities, survivors and potential perpetrators understand the nature of violence. Survivors make decisions, potential perpetrators think again, and other people understand both the nature of violence and that action is possible. People stand up against violence against women and girls, individually and collectively, and community members think and act to help survivors. Families and communities stop accepting violence and strengthen community structures that support a conviction that it is intolerable. Institutional support from non-government organisations, the police, medical practitioners, and lawyers is accessible and functional and families and communities free themselves of violence against women and girls.

In this formulation, community mobilisation has two general aims: primary prevention through development of awareness of the importance and iniquity of violence against women, accompanied by knowledge of rights and law, and secondary prevention through increased identification of survivors and individual and collective action to support them. Prevention encompasses a range of interventions aiming to reduce risks or threats to health and wellbeing, grouped into three categories: primary, secondary, and tertiary. We believe that all three – particularly primary and secondary prevention - lead to reduction of violence at individual, relationship, and community levels. Primary prevention is exemplified by previous trials in which interventions have focused on increasing community awareness of violence against women, inequitable gender norms, and women’s rights. Primary prevention not only targets specific causes and risk factors for violence against women and girls, but also aims to promote healthy behaviours, increase knowledge of rights and entitlements, and improve women’s capacity to resist violence.

Secondary prevention usually describes interventions to support women survivors of violence in order to prevent it continuing or mitigate it. We see the enactment of these interventions as a means of both highlighting violence in the community and showing that redress and resolution are possible. In this sense, it speaks to the modelling function within the COM-B framework (a product of education, persuasion and coercion). Visible intervention to support survivors makes people aware of the problem and potential solutions. This helps to foster safe environments that reduce the risk of violence; for instance, through creating networks that offer support to women. Intervening in emergencies and demonstrating action builds trust and confidence, increases awareness and knowledge in communities, and reduces community members’ tolerance of violence. Tertiary intervention describes interventions to support women in dealing with the consequences of violence, particularly effects on their mental health. Again, the pathway of support, from community activist to counsellor to psychologist and psychiatrist, sends a message to other women in difficult circumstances.

Overall, the theory envisages counsellors, community mobilisers, community volunteers and groups of women, men, and youth working together to bring about individual and collective change with the ultimate aim of reducing domestic violence. The theory suggests that community mobilisation encourages transformation of participants and pro-social action prevents violence against women and girls, as well as bystander intervention, while local support and response, crisis counselling, medical, psychosocial, police and legal support contribute to the identification and support of survivors of violence. Visible instances of support and justice for survivors are thought to encourage greater community activism, thus completing a positive feedback loop between primary prevention (through community mobilisation) and secondary prevention (through institutional support for survivors). The detailed steps are described below.

### Resources and interventions

First, the program inputs comprise salaried counsellors, community organisers, community officers, and coordinators, as well as voluntary human resources from women, men, and young people who join groups. Table 2 summarises expected roles for each type of individual, keyed numerically with Figure 1 in three categories. Mobilisation is primarily the remit of community organisers who identify potential group members, bring them together, and facilitate a sequence of modules and discussions. Group sessions aim to build a political agenda for change that privileges women’s interests and looks to transform gender and social power relations. Our group-work module follows a sequence over three years. The first year emphasises awareness of violence, gender norms, women’s rights and entitlements, and the importance and strength of women’s collectives in addressing violence against women and girls. The second year emphasises action, be it individual or collective. The third year emphasises leadership and group members are encouraged to take on prominent roles in the community. Generally, women who are proactive, articulate, have a social network in their neighbourhoods, and are willing to devote time to the issue are recommended as volunteer activists by groups and invited to become *sanginis*.

Along with group members, community organisers also identify survivors of violence and help group members to respond. Organisers are initiators and points of contact for involvement of counsellors and follow-up with survivors. They facilitate community events and campaigns and liaise with medical, police, and legal services. Counsellors are based close to communities and conduct crisis intervention, counselling, and home visits, as well as providing institutional support. Group members become involved in campaigns and identify and support survivors of violence. After about one year of group activity, *sanginis* emerge and are trained to identify and support survivors, as well as in communication and liaison with services. Community organisers work closely with *sanginis* whom they identify and mentor.

### Changes

Second, the theory proposes three forms of local change: an increase in identification of and support for survivors of violence, changes in the beliefs and actions of group participants and volunteer *sanginis*, and broader changes in communities. Survivors are able to view their experience in a broader context, get help when they feel they need it, and take action to improve their situation. This visible secondary prevention leads to community awareness, increased identification and consultation, and a broad base of support. Participation in groups leads to changes in members and awareness of gender issues. This yields action in terms of individual and collective efficacy based on precedent, and visibility in the community for groups and individuals who take on leadership and volunteer roles.

### Outcomes

Third, group and individual activities, linked with tangible service provision and successful interactions with counsellors, the police, and lawyers, lead to increased disclosure of violence. We believe that changes in attitudes are more likely to follow changes in behaviour than the other way around. Although there is a place for awareness and attitudinal change, our programmatic experience suggests that changes in social norms and attitudes can be accelerated by visible instances of successful response to the needs of survivors of violence. These responses themselves reduce the prevalence of domestic violence through secondary prevention, but the accompanying awareness and belief in rights and recourse is a form of primary prevention. Our main emphasis is on domestic violence, but it is conceivable that changes in community norms and bystander intervention will also reduce the likelihood of non-partner sexual violence outside the home.

### Undesirable changes

Finally, the intervention might lead to a number of changes for the worse in homes and communities ( Chen & Rossi, 1980). These are based on program experience and have been called ‘dark logic’ in the context of program theory ( Bonell *et al.*, 2015). Community interventions might lead to an increase in violence against women and girls as gender norms are transgressed and people push back against existing controls on women’s behaviour. This could be a short-term negative effect. Conversely, growing opposition to violence might lead to vigilantism and precipitate action and punishment meted out to either survivors or people who were not perpetrators. For example, after a group session on ration rights, a men’s group member called for a violent protest. Awareness of the problem of non-partner sexual violence might lead families to set limits to women’s mobility, and awareness of community and legal sanctions might lead perpetrators to modify the kind of violence they use. As concerns surface, it is conceivable that group members, volunteer *sanginis*, community organisers, counsellors, or the families of survivors of violence might face threats or exclusion. At one point, ration shopkeepers threatened a sit-in outside our organisation’s office because of our help with complaints. The unexpectedly positive effect of this negative response was that more people volunteered to join the organisation. Finally, the program’s focus on personal development and leadership might support people with personal agendas not entirely aligned with its aims, or might lead to favouritism. An example is a case in which a women’s group member stood out as a leader. She started her own group, which undertook several successful actions, but took community action on herself and would not allow others to lead. Eventually, community members would not participate in an electricity campaign and when she moved out of the area nobody took her work forward.

### General theory

Three areas of disciplinary theory meet in the ideas that underlie our program theory: social norms theory (already prominent in prevention of violence against women), network theory (prominent in political science, economics, and latterly in public health), and behaviour change theory (prominent in behavioural psychology and public health).

Our ideas about norms are informed by integrated theory with a feminist perspective ( Heise, 1998), which also underlies our organizational approach to working with survivors of violence. Beyond the need to work with families, we aim to ensure that no further harm is done to women, who remain the focus and whose views and making of meaning are prioritized. We believe that violence against women deserves attention as a gendered phenomenon beyond family violence ( Lawson, 2012), and that gender needs to be in the foreground ( Dobash & Dobash, 1979; Gelles, 1985). Our attempts to achieve gender transformative change are particularly indebted to theory around asymmetric distribution of power between genders ( Rodrígues-Menés & Safranoff, 2012), and hegemonic patriarchy ( Connell, 1987; Connell, 1995).

Our thinking on potential mechanisms has been influenced by social norms theory, and particularly by discussions of the need to transform gender norms as a route to addressing violence against women ( Alexander-Scott *et al*., 2016). This is particularly relevant because we are concerned about the forms of violence that constitute gender-based household maltreatment: emotional and economic violence, control and neglect. Our previous work suggested that we might focus on three ideas about norm change ( Daruwalla *et al*., 2017). The first is to make use of the mismatch between descriptive and injunctive norms. A descriptive norm describes beliefs about what other people do ( Bicchieri, 2006; Cialdini *et al*., 1991; Muldoon *et al*., 2014), while an injunctive norm describes beliefs about what other people think one ought to do ( Bicchieri, 2006; Cialdini *et al*., 1991). Descriptive norms intolerant of violence are likely to be magnets for behaviour and if we are able to show examples of non-violent interaction we might be able to influence people’s perception of what is actually prevalent in the community. This is helped by our second idea, which is that injunctive norms disapprove (at least in principle) of violence. How to spread awareness of this turns on the reference group - the people surrounding an individual from whom she takes her cue ( Levy Paluck *et al*., 2010) - and our third idea is to expand the currently limited reference groups around individuals so that they can process a larger pool of opinion on norms intolerant of violence.

When we ‘expand a reference group’, we are basically saying that individuals meet and engage with more people, and this evokes ideas from network or social capital theory ( Putnam, 1995). Another way of seeing the new connections between people that develop in community groups is as weak ties or bridging social capital, in contradistinction to the (hierarchical) strong ties or bonding social capital that the family circle typifies ( Granovetter, 2005; Patulny & Svendsen, 2007). Added to this is the idea of linking social capital between, for example, community members and institutions that might help them to address violence against women ( Szreter & Woolcock, 2004), a process explicit in our theory of change. So, although community mobilisation is a way to address violence against women, it can be seen equally as a community building or social capital endeavour (see ( Szreter & Woolcock, 2004) for an anatomisation of the debates around social capital and public health).

We use the Capability, Opportunity, and Motivation model (COM-B) to think about individual behaviour change, particularly in terms of the functions of interventions that it summarises ( Michie *et al*., 2011). The program aims to increase the capability of participants to understand violence against women, identify survivors, and take supportive action, with knowledge of law and rights, decision-making and negotiation skills, self-efficacy, and collective efficacy. This it does predominantly through education, training, enablement, and modelling (first by program staff and then by community members). It presents opportunities to engage with others, learn, develop confidence and leadership, and connect with supportive non-government and government organisations; again predominantly through education, training, enablement, and modelling. Motivation comes from the development of belief in preventing violence against women and children, increased by education, persuasion, enablement, training, and modelling. From the point of view of potential perpetrators of violence, the primary functions are persuasion, coercion, and education, accompanied (we hope) by awareness and change in attitude brought about by education and modelling and reduced opportunity for violent behaviour.

## Discussion

Our theory of change differs from existing theories of change in a number of ways: it is adapted for the program’s context; it was designed through an extended consultative process from 2015 to 2017; it places major emphasis on secondary prevention as a pathway to primary prevention; it integrates community activism with referral and counselling interventions; and it makes explicit specific testable causal pathways to impact, which will be evaluated within the context of an on-going cluster-randomised controlled trial. While some previous theories of change share some of these characteristics, to our knowledge, no previous theory has had them all.

This article describes the theory of change behind a comprehensive community-based intervention to prevent violence against women through primary and secondary prevention. It took 22 months to develop the theory and involved primary data collection with multiple stakeholders, multiple workshops with critical commentators, and many team meetings. This long and careful process of theory building has resulted in a theory that improves on existing theories of change for the prevention of violence against women in a few ways.

First, the theory highlights the interconnectedness of primary and secondary prevention through a positive feedback loop. Community members become capable and motivated to identify and refer survivors to crisis counselling and institutions; in turn, successful resolution of cases of violence with institutional actors - non-government organisations, the police, medical practitioners, and lawyers - raise awareness and strengthen community members’ confidence in their own activism. Community activism still takes place through individual outreach, group discussion and reflection, or community-wide campaigns, but it is closely linked to support from local counselling and legal aid centres. This model contrasts with previous theories of change, which have tended to place their main emphasis on primary prevention through community activism and capacity building to develop awareness of burden, rights, law, and recourse ( Abramsky *et al.*, 2016; Falb *et al.*, 2016; Pettifor *et al.*, 2015; Wagman *et al.*, 2015).

Second, the theory was fully adapted to the local context of urban informal settlements in India, while previous theories of change have predominantly been developed for a Sub-Saharan African context ( Abramsky *et al.*, 2016; Falb *et al.*, 2016; Pettifor *et al.*, 2015; Wagman *et al.*, 2015) with the notable exception of a single study in Nepal ( Clark *et al.*, 2017). Many elements of the current theory of change reflect previous experience over the past 15 years of program activity.

Third, the theory provides greater specificity than previous theories. These can broadly be categorised into three types: quasi-linear logic models ( Clark *et al.*, 2017; Wagman *et al.*, 2015), stages of change models ( Abramsky *et al.*, 2014; Falb *et al.*, 2014; Michau, 2007), and ecological models ( Abramsky *et al.*, 2016; Michau *et al.*, 2015). Ecological models tend to see violence reduction as arising from the simultaneous operation of a large number of activities and processes at individual, household, and community levels which interact in unspecified ways. Stages of change models view violence prevention activities as progressing in stages from community entry to awareness-raising to behaviour change, but do not always specify why or how communities progress from one stage to the next. Quasi-linear logic models present intervention processes as a block of activities leading via a block arrow to another block of changes and outputs. Such models often lack clarity on the exact ‘context-mechanism-outcome configurations’ ( Pawson & Tilley, 1997) that are expected to occur. The current theory of change lists the pre-conditions that need to be fulfilled for each component of the theory to ‘work’, the causal connections between each component, as well as any adverse effects that may arise. A similar approach has been taken in the development of Sonke CHANGE, a cluster randomised controlled trial in periurban Johannesburg, South Africa. The Sonke Gender Justice intervention involves workshops and community action teams working predominantly with men with an emphasis on changing harmful gender norms and prevailing hegemonic masculinity. The program theory of change proposes that community action and advocacy to promote equitable gender norms and non-violent masculine attitudes and practices will lead to enhanced critical consciousness, collective efficacy and action, and better social cohesion and trust. These will lead in turn to self-efficacy to take action, reduced influence of harmful gender norms, and an enabling environment for policy implementation ( Christofides *et al*., 2018).

Theories of change are necessarily provisional, and may not be right, but they still serve useful functions as guides to evaluation ( Birckmayer & Weiss, 2000). By providing a theoretical framework for collecting and analysing data, they can help overcome problems intrinsic to “omnibus data” ( Auspos & Kubisch, 2004) that are insufficiently directional to test theory ( Weiss, 1997). The current theory of change has allowed us to understand our program, consider the necessity of specific components, and be specific about intermediary changes ( Birckmayer & Weiss, 2000). It also helped to clearly articulate program objectives across a range of team members—community organisers, qualitative and quantitative evaluators, anthropologists, economists, medical practitioners, psychologists, and legal advisors—with stakes in the program ( Mason & Barnes, 2007). In turn, this has helped to draw lessons from experience, conduct strategic planning, communicate the working of SNEHA’s program to other people, and select outcomes and indicators for monitoring and evaluation.

We are currently doing a cluster randomised controlled trial of a scalable set of interventions, specifying components and evaluating effectiveness in direct response to the theory of change. Previous evaluations have presented program theories which were subsequently only partially used for evaluation purposes. For example, many individual proposed mediators of intervention effect remain untested. Changes in policymakers, community leaders, and professionals’ attitudes, knowledge and beliefs about violence against women and girls are often hypothesised as mediators of intervention effect, but they have rarely been measured or reported ( Abramsky *et al.*, 2016; Wagman *et al.*, 2015). Similarly, policy change at national or sub-national levels is often included in the theory of change, but excluded in the intervention impact evaluation ( Abramsky *et al.*, 2016; Clark *et al.*, 2017; Wagman *et al.*, 2015). For example, Falb *et al.* (2016) hypothesised that their intervention would increase the human, social, physical, and financial assets of girls, but focused on human and social assets in their outcome evaluation. A recent review of 62 studies of theory-informed evaluation in public health noted that integration of theory into randomised controlled trials was often limited ( Breuer *et al.*, 2016). We intend to measure and evaluate all aspects of our theory of change in our cluster randomised controlled trial.

A potential criticism of a theory of change is that the connections between specific activities and outcomes are often insufficiently understood, a challenge that we call the *block and arrow* problem. Given our theory’s provisional nature, we hope to open up the blocks to understand the ways in which their components ‘work’. This is clearly difficult in a complex system with multiple inputs and emergent phenomena, but can be divided into three projects. The first project involves the stuff of process evaluation: to understand what was delivered, how well, what was received, and by whom. An evaluation framework is in place to address these questions both qualitatively and quantitatively in terms of reach, efficacy, adoption, implementation, and maintenance (the RE-AIM framework ( Glasgow *et al*., 1999), which we have used before ( Shah More *et al*., 2013)). The second project is to understand whether each of the components of the three boxes describing change happens. We hope to evaluate this quantitatively through an electronic intervention monitoring system and simultaneous qualitative observation and interview: each of the changes listed in the boxes has been taken as an indicator. For example, we will track bystander intervention quantitatively and through case studies. We will enumerate identification of survivors and their subsequent communication with medical, police, and legal services. We will conduct qualitative and quantitative assessments of people’s understanding of violence and the degree to which it ceases to be thought of as a private family matter. We will follow group membership, actions taken by members, and the development of leadership. And we will document community enquiries and referrals as a result of violence against women.

The third project is to understand the mechanisms through which program effects are achieved. Like many public health implementors, we have a strong interest in realist evaluation ( Pawson & Tilley, 1997). Within an intervention with many moving parts, which activities work well, for whom, and in which contexts? Our aim is to generate candidate context-mechanism-outcome configurations that we can propose as hypotheses, and to test them. The context of program sites will be described after a combination of ethnographic work, participatory learning and action activities, and quantitative data collection on demography, sociocultural indices, and experience of and response to violence against women. We take a method-neutral approach, combining ethnographic interviews and observation, targeted qualitative interviews with staff, group members, and volunteers, and case studies of how the intervention unfolds in selected localities; these augmented with data from our intervention monitoring system that can be used to answer specific questions. Central issues for consideration include the contribution of secondary prevention to primary prevention, the role of collective action ( Gram *et al*., 2019), the role of expanded reference groups in norm change, and the role of men in program effectiveness ( Chakraborty *et al*., 2018).

An interesting challenge to the development and use of our theory of change has been understanding how to frame work that has traditionally taken a feminist social position within the developing public health paradigm for complex interventions. Sociologists have proposed that randomised controlled trials could be used hypothetico-deductively to test and refine theories of change for complex public health interventions ( Bonell *et al.*, 2018). Coryn and colleagues have proposed five principles for theory-driven evaluation. It should (1) formulate a plausible program theory, (2) formulate and prioritise evaluation questions around the theory, (3), be used to guide planning, design, and execution of the evaluation, (4) measure constructs postulated in the theory, and (5) identify breakdowns and side-effects, determine program effectiveness or efficacy, and explain cause and effect associations between theoretical constructs ( Coryn *et al.*, 2011). These principles sometimes fit awkwardly with feminist concerns with building an activist social movement rather than a managerial, professional organisation. We are trying to do both.

## Conclusion

20 years ago, Weiss said that, “If theory is taken to mean a set of highly general, logically interrelated propositions that claim to explain the phenomena of interest, theory-based evaluation is presumptuous in its appropriation of the word. The theory involved is much less abstract and more specific, more selective, and directed at only that part of the causal chain of explanation that the program being evaluated is attempting to alter” ( Weiss, 1997). This certainly applies to several existing theories for prevention of violence, which include macro concerns such as national development, sectoral issues, organisational aims, or program aims ( James, 2011), and whose lack of specificity has made it hard to apply them to the current theory of change.

We are particularly struck by the rapidity with which draft theories are often developed. The field tends to quite quickly adopt new approaches and there is a danger that, if theory of change is seen as something that can be produced after two or three variably attended workshops ( Gooding *et al.*, 2018), its undoubted benefits will be seen as a fad. Again, the assumption is that theory will be revisited and amended ( Mason & Barnes, 2007), but program realities—and perhaps a lack of time and space to interrogate assumptions ( Archibald *et al.*, 2016)—conspire to make this uncommon.

We hope that our theory of change is plausible, feasible, and testable ( Kubisch, 1997). In developing it, we had four advantages: sufficient time, 15 years of program activities to examine, evaluators as core team members, and stakeholder involvement ( Auspos & Kubisch, 2004). This meant that we were able to ensure that the theory fitted the context and the opinions of diverse contributors ( Moore & Evans, 2017). We hope that our theory will become a useful tool for researchers, practitioners and policy-makers working in similar contexts to think through the pathways through which they hope to achieve impact on violence against women.

## Data availability

### Underlying data

UK Data Service: Changing gender norms in the prevention of violence against women and girls in India. https://doi.org/10.5255/UKDA-SN-852735 ( Osrin, 2017).

This project contains transcripts of focus group discussions and interviews, translated into English. The safeguarded data files are made available to users registered with the UK Data Service under UK Data Archive End User Licence conditions. The data files are not personal, but—given the subject matter of the interviews and focus groups—the data owner and research ethics committee consider there to be a limited residual risk of disclosure.

### Extended data

Open Science Framework: A theory of change for community interventions to prevent domestic violence against women and girls in Mumbai, India. https://doi.org/10.17605/OSF.IO/47JMG ( Osrin, 2019).

This project contains the following extended data:

- Action_documentation_archive.xlsx
- Consultant_report_2016.docx (initial consultancy report)
- Reference_list.docx (reference list for development of theory of change)
- ToC_development_history.pdf (theory of change visual development history)
- ToC_meetings_summary.docx (theory of change meetings summary)

Extended data are available under the terms of the Creative Commons Attribution 4.0 International license (CC-BY 4.0).
