# Supplementary material for: A theory of change for community interventions to prevent domestic violence against women and girls in Mumbai, India
Source: Wellcome Open Res. 2019 Aug 21;4:54. Originally published 2019 Mar 25. [Version 2] doi: 10.12688/wellcomeopenres.15128.2 (PMC6719749; doi:10.12688/wellcomeopenres.15128.2)
Supplement: Peer review history

# Peer review: Reviewer response for version 2

**DOI:** 10.21956/wellcomeopenres.16844.r36281

**Published:** 2019-08-30

**Authors:** Abigail M. Hatcher

**Affiliations:** 1 Department of Medicine, University of California, San Francisco (UCSF), San Francisco, CA, USA; 2 School of Public Health, University of the Witwatersrand, Johannesburg, South Africa

I have no additional comments and felt the authors responded fully to the first review.

I confirm that I have read this submission and believe that I have an appropriate level of expertise to confirm that it is of an acceptable scientific standard.

# Peer review: Reviewer response for version 2

**DOI:** 10.21956/wellcomeopenres.16844.r36280

**Published:** 2019-08-29

**Authors:** Angela Taft

**Affiliations:** 1 Judith Lumley Centre, La Trobe University, Bundoora, Australia

While I might quibble with the positioning of the description of range of general theories after the outline of the methods used in this theory development, I am comfortable that this paper deserves to be indexed and I look forward to reading the trial outcomes.

 I think the argument about the need for evidence informing prevention theory on VAW that takes into account the interdependence of actions at community level that are secondary responses but that may have preventive consequences or cause harm is valid and important.

 An important study in a small field in LMI countries.

I confirm that I have read this submission and believe that I have an appropriate level of expertise to confirm that it is of an acceptable scientific standard.

# Peer review: Reviewer response for version 1

**DOI:** 10.21956/wellcomeopenres.16507.r35652

**Published:** 2019-06-17

**Authors:** Angela Taft

**Affiliations:** 1 Judith Lumley Centre, La Trobe University, Bundoora, Australia

This theory development and the impressive amount of empirical work preceding has the potential to make a substantial contribution to a very limited literature of prevention interventions, as it aims to develop and test a theoretical model of feedback between primary and secondary prevention in a low-income and patriarchal society. It is well written and structured. It is part of an ambitious and impressive set of studies over many years by the team at SNEHA.

 I have articulated below where I think your outline could be clarified for improved understanding by readers. My comments echo those of Abigail Hatcher in suggesting that more clarity is needed about the mechanisms of action between all three phases of the model.

**Introduction**

 Excellent and clear rationale for the study and the need for theory in a very challenging environment for an IPV prevention program.

1. I urge you to define how you understand primary and secondary prevention and apply it to your work, especially to clarify your intentions in the penultimate paragraph prior to the Methods section. For example, you mention both in the second paragraph of page 3, but it is not clear to which category the activities you describe belongs.
2. P.3 Second column, second paragraph. You talk about your cadre of volunteers who take on special responsibilities. I suggest you name them as ' **sanginis**', as I remained puzzled as to what their responsibilities and roles were until you defined them only in the top paragraph on page 9, which is too late.

**Methods**

 You mostly clearly present the set of strategies used to develop your emerging theory of change.

1. p.4  - 21% of ever-married women have experienced IPV – ‘ever’ or in previous twelve months?
2. It would be helpful to describe what characterised the actions at each ecological level you describe, and I agree with the first reviewer about the need to understand how these were selected for Table 1.
3. In Table 1, why were your actions not included under these category levels which would have been more useful than below types of violence, given your decision to focus on domestic violence against women and girls? Why is non intimate partner violence and child abuse included in this table if it is part of your theory building exercise?
4. Ethical approval – can you please provide more information about Ethicos, given that searches for them do not reveal much about their scope and practice.
5. How was the literature review incorporated with your previous work and who had access to its findings. How did it contribute to your findings, given it was your last undertaking?

**Results**

1. Primary and secondary intervention are now defined for the first time. Again, if you have decided on domestic violence against women and girls only, why is non-partner sexual violence (very important but different) prevalence included in Figure 1 - Theory of change?
2. It is not clear to me how institutions such as the health care system are involved or mobilised, although their crisis responses are critical to the responses. How are they theorised into this model? It is only mentioned in passing on page 9 at the end of the paragraph on outcomes.
3. Please clarify what ‘institutional support’ means in the first paragraph on Page 9.
4. I also congratulate the investigators for their attention to ‘dark logic’ - potential adverse effects, but I would like more discussion about how they were predicted and managed, as there are considerable and real dangers to your workers illustrated in Table 1. You address a description of what you found or could anticipate in ‘Undesirable changes' on page 9 – but you do not theorise how you might address it in your prevention theory.

**Discussion**

 You argue that the theory has a strong empirical basis in their previous programs of work, is adapted to the South Asian (Indian) context rather than Africa and posits a feedback loop between secondary and primary prevention. This is admirable and indeed innovative and it will be interesting to read the outcomes of the randomised trial that is proposed to ‘measure and evaluate all aspects of our theory of change’.

 However, you also argue that your current theory ‘lists pre-conditions that need to be fulfilled for each component to work’. Without comprehensive process evaluation for the interim stages between the resources – changes – outcomes sections of the theory (for example above of effective training programs or organisational links), the proposition that these demonstrate causal pathways appears very speculative.

 Indeed your interim stages of training and building strong and essential individual, team and organisational capacity and these process outcomes or levels of quality are missing stages in the theory development and really need to be better addressed.

I confirm that I have read this submission and believe that I have an appropriate level of expertise to confirm that it is of an acceptable scientific standard, however I have significant reservations, as outlined above.

# Response

**Published:** 2019-08-11

**Authors:** David Osrin

**Affiliations:** University College London, UK

Thanks to both reviewers for their enthusiasm for the paper and very helpful suggestions.

*Comment 1*

My comments echo those of Abigail Hatcher in suggesting that more clarity is needed about the mechanisms of action between all three phases of the model.

*Response*

We hope that the additions presented in response to Abigail Hatcher go some way toward clarity.

*Comment 2*

I urge you to define how you understand primary and secondary prevention and apply it to your work, especially to clarify your intentions in the penultimate paragraph prior to the Methods section. For example, you mention both in the second paragraph of page 3, but it is not clear to which category the activities you describe belongs.

*Response*

We have added to the text in the Results.

“In this formulation, community mobilisation has two general aims: primary prevention through development of awareness of the importance and iniquity of violence against women, accompanied by knowledge of rights and law, and secondary prevention through increased identification of survivors and individual and collective action to support them. Prevention encompasses a range of interventions aiming to reduce risks or threats to health and wellbeing, grouped into three categories: primary, secondary, and tertiary. We believe that all three – particularly primary and secondary prevention - lead to reduction of violence at individual, relationship, and community levels. Primary prevention is exemplified by previous trials in which interventions have focused on increasing community awareness of violence against women, inequitable gender norms, and women’s rights. Primary prevention not only targets specific causes and risk factors for violence against women and girls, but also aims to promote healthy behaviours, increase knowledge of rights and entitlements, and improve women’s capacity to resist violence.”

“Secondary prevention usually describes interventions to support women survivors of violence in order to prevent it continuing or mitigate it. We see the enactment of these interventions as a means of both highlighting violence in the community and showing that redress and resolution are possible. In this sense, it speaks to the modelling function within the COM-B framework (a product of education, persuasion and coercion). Visible intervention to support survivors makes people aware of the problem and potential solutions. This helps to foster safe environments that reduce the risk of violence; for instance, through creating networks that offer support to women. Intervening in emergencies and demonstrating action builds trust and confidence, increases awareness and knowledge in communities, and reduces community members’ tolerance of violence. Tertiary intervention describes interventions to support women in dealing with the consequences of violence, particularly effects on their mental health. Again, the pathway of support, from community activist to counsellor to psychologist and psychiatrist, sends a message to other women in difficult circumstances.”

*Comment 3*

P.3 Second column, second paragraph. You talk about your cadre of volunteers who take on special responsibilities. I suggest you name them as 'sanginis', as I remained puzzled as to what their responsibilities and roles were until you defined them only in the top paragraph on page 9, which is too late.

*Response*

Thanks. We’ve done this.

*Comment 4*

Methods. You mostly clearly present the set of strategies used to develop your emerging theory of change. p.4  - 21% of ever-married women have experienced IPV – ‘ever’ or in previous twelve months?

*Response*

It is ‘ever’ and we have clarified this in the text.

*Comment 5*

It would be helpful to describe what characterised the actions at each ecological level you describe, and I agree with the first reviewer about the need to understand how these were selected for Table 1. In Table 1, why were your actions not included under these category levels which would have been more useful than below types of violence, given your decision to focus on domestic violence against women and girls? Why is non intimate partner violence and child abuse included in this table if it is part of your theory building exercise?

*Response*

We have redrafted Table 1, added columns, and added text to the Methods. Please see our response to Abigail Hatcher, and we have added the following to the Methods:

“Although our commitment is to preventing domestic violence, both individual and collective actions often responded to incidents in the neighbourhood. Different kinds of violence – and particularly sexual violence in public spaces – coexist and response to each is both a source of confidence and a step toward a belief in collective efficacy. Likewise, groups often undertook collective action to improve their environment and we see this as contributing to a sense of collective efficacy and community building.”

*Comment 6*

Ethical approval – can you please provide more information about Ethicos, given that searches for them do not reveal much about their scope and practice.

*Response*

The Ethicos Independent Ethics Committee is registered with the Central Drugs Standard Control Organization, Directorate General of Health Services, Ministry of Health & Family Welfare, Government of India. It includes physicians, psychologists, and other healthcare professionals. The ethics committee is organised and operates according to the requirements of the Indian Council of Medical Research, Good Clinical Practice, local law, and regulatory requirements.

*Comment 7*

How was the literature review incorporated with your previous work and who had access to its findings. How did it contribute to your findings, given it was your last undertaking?

*Response*

Apologies for the lack of clarity. We were reviewing literature throughout the theory building process but did not conduct a formal systematic review until the end. The purpose of the review was in part to uncover theoretical gaps we had not considered in our framework, but we did not find any major gaps.

*Comment 8*

Results. Primary and secondary intervention are now defined for the first time. Again, if you have decided on domestic violence against women and girls only, why is non-partner sexual violence (very important but different) prevalence included in Figure 1 - Theory of change?

*Response*

We have added information about our inclusion of non-domestic violence to the Methods (response to Comment 5). Managing community expectations is critical for programs to prevent violence against women. One of the principles of community organisation and mobilisation is to work on issues that community members prioritise. Consciousness raising is an important strategy in our prevention work. Violence against women and girls is pervasive, ubiquitous, and multiform and our program seeks to build a holistic understanding. Although domestic and intimate partner violence are the commonest reasons for consultation at our counselling centres (and are our priority), women face other forms of violence in their communities. For example, during the microplanning process, women consistently report non-partner sexual violence in their families and outside their homes. It is difficult to segregate interventions on different forms of violence when we expect communities to take over and build strategies to prevent it. Moreover, community members are more interested in intervening in non-partner sexual violence as this is not considered to be a private matter and everyone has a stake in it.

*Comment 9*

It is not clear to me how institutions such as the health care system are involved or mobilised, although their crisis responses are critical to the responses. How are they theorised into this model? It is only mentioned in passing on page 9 at the end of the paragraph on outcomes.

*Response*

We hope that the revised version makes this clear. Health system responsiveness can be seen on a continuum of prevention and response in in addressing violence, including recognition and voicing of the issue, identification of vulnerabilities, facilitating referrals and institutional linkages, and creating an enabling environment. The tertiary initiatives entail counselling and legal service, advocacy for burn survivors and sensitization of staff, referrals, counselling and legal services, and ensuring deputation for training and provision of space. Tertiary prevention can be enhanced if linkages are built between the health care system and community members to monitor cases of violence, ensure long-term medical treatment in cases of mental ill health and extend support by the community as and when required.

*Comment 10*

I also congratulate the investigators for their attention to ‘dark logic’ - potential adverse effects, but I would like more discussion about how they were predicted and managed, as there are considerable and real dangers to your workers illustrated in Table 1. You address a description of what you found or could anticipate in ‘Undesirable changes' on page 9 – but you do not theorise how you might address it in your prevention theory.

*Response*

In restructuring Table 1, we have removed the information on adverse events and placed it in the Results under *undesirable changes*. These were based largely on experience over the years. Although response to each eventuality will differ and it may, as the examples included in the section now show, have an unpredicted positive outcome, it is always based on three principles: the safety of both survivors of violence and team members, organisational support, and adherence to protocols. A range of protocols are in place for response to threat and crisis, and field team members are mandated to call for support from experienced managers in any eventuality. A recent example is an incident in which local individual men became aware that women were discussing violence with our team and immediately called for suspension of activities. Senior managers met with the individuals involved, local women, and the sitting political representative - with support from the police – and the situation was clarified and resolved to the satisfaction of all parties. A second example from some years ago involved our activities being opposed by local community members after assistance for a survivor of child sexual abuse. This was resolved after community discussions with senior managers and program activities have continued in the area to date.

*Comment 11*

Discussion. You argue that the theory has a strong empirical basis in their previous programs of work, is adapted to the South Asian (Indian) context rather than Africa and posits a feedback loop between secondary and primary prevention. This is admirable and indeed innovative and it will be interesting to read the outcomes of the randomised trial that is proposed to ‘measure and evaluate all aspects of our theory of change’.

However, you also argue that your current theory ‘lists pre-conditions that need to be fulfilled for each component to work’. Without comprehensive process evaluation for the interim stages between the resources – changes – outcomes sections of the theory (for example above of effective training programs or organisational links), the proposition that these demonstrate causal pathways appears very speculative.

Indeed your interim stages of training and building strong and essential individual, team and organisational capacity and these process outcomes or levels of quality are missing stages in the theory development and really need to be better addressed.

*Response*: These comments are well taken and we have added material to Results and Discussion sections. Please see our response to Abigail Hatcher.

# Peer review: Reviewer response for version 1

**DOI:** 10.21956/wellcomeopenres.16507.r35121

**Published:** 2019-04-30

**Authors:** Abigail M. Hatcher

**Affiliations:** 1 Department of Medicine, University of California, San Francisco (UCSF), San Francisco, CA, USA; 2 School of Public Health, University of the Witwatersrand, Johannesburg, South Africa

This manuscript was a joy to read and addresses an issue sorely under-explored in the violence prevention literature: how to design programs so that the underlying theory is testable. To be fair, the prevention of intimate partner violence is also lacking efficacious interventions (with or without theoretical underpinnings). Yet, if new projects can be developed with strong theories of change articulated alongside them, it is feasible for outcomes to be achieved alongside a consistent advance of the theory. This is laudable and I am grateful for the hard work the authors have led towards this goal.

 I have a few comments that may inform future efforts for this team and for others aiming to prevent intimate partner violence.

1. How were the actions detailed in Table 1 chosen from among the 76 total actions documented by the community mobilization team? Were these coded thematically, and was that coding undertaken by a single researcher or multiple team members? How was consensus reached and discrepancies resolved? The “Action type” and “Intervention function” are interesting but these do not map onto the theory of change, as far as I can tell. A better description of these categories and what they mean would be valuable.
2. The theory of change itself in Figure 1 adds considerably to the literature. I am particularly impressed by the assumptions that are articulated. However, I’m concerned that the arrows leading from “Resources” to “Changes” seem to be, in and of themselves, something of a black box. I would like to know (and the IPV field is search for answers to the question of) *how *community volunteers and groups of women and men work together to change participants? Indeed, how do these volunteers and community members take up their work in such a way that communities change overall? Is it through solidarity and group mutual support? Or is it through personal change that leads to an overall community-level benefit (i.e. the whole is greater than the sum of its parts)? The mechanism of action is not identified, which makes it very challenging to test the underlying assumptions of the theory. One suggestion would be to highlight important Theory (as in, sociological or behavioral theory) either within the figure or the accompanying text.
3. The notion of a “ceiling of accountability” is interesting, and I wonder if the authors can discuss how many IPV prevention programs position themselves along these lines. While many programs cite their stated aim is to reduce domestic violence, few projects globally have ever proven that they are able to do this.
4. It would be nice to hear a bit more about how *sanginis* “emerge” (or are chosen by the program to take up this role).
5. The section on “Undesirable changes” is excellent and crucial for the field, though this is the first time I have seen these concerns articulated so thoughtfully.
6. As above, the discussion statement around “makes explicit specific testable causal pathways to impact” is not entirely right, since the pathways themselves are poorly fleshed out. While the ‘context’ and ‘outcome’ configurations are indeed mapped out, I believe the ‘mechanism’ linking the two requires additional thinking.
7. I agree with the authors position that this is among the first projects to meaningfully combine primary and secondary prevention, and this is also crucial for the field.
8. The protocol by Christofides et al (2016) ^1^ would be important to include in the discussion, given its emphasis on a theory of change for primary prevention of IPV in a similar peri-urban setting. This would be particularly valuable in the sections on the three types of theory and the section about how policy change is incorporated into a theory of change and the concomitant impact evaluation.
9. What methods beyond running a trial are the authors engaged with presently? For example, will they harness path analysis to unpack the mechanisms underlying intervention success? Will they conduct a qualitative process evaluation alongside the trial?
10. While the contradictions between feminist framing and the needs of trials/projects to be managerial in nature is important, I wonder if it could use additional thinking. In particular, this is an area where Theory (capital T) could be brought forward more intentionally, since the authors are clearly drawing from multiple approaches and epistemologies.

I confirm that I have read this submission and believe that I have an appropriate level of expertise to confirm that it is of an acceptable scientific standard, however I have significant reservations, as outlined above.

## References

[1] : A cluster randomised controlled trial to determine the effect of community mobilisation and advocacy on men’s use of violence in periurban South Africa: study protocol. *BMJ Open*.2018;8(3) : 10.1136/bmjopen-2017-017579 10.1136/bmjopen-2017-017579 PMC587565129574438

# Response

**Published:** 2019-08-11

**Authors:** David Osrin

**Affiliations:** University College London, UK

Thanks to both reviewers for their enthusiasm for the paper and very helpful suggestions.

*Comment 1*

How were the actions detailed in Table 1 chosen from among the 76 total actions documented by the community mobilization team? Were these coded thematically, and was that coding undertaken by a single researcher or multiple team members? How was consensus reached and discrepancies resolved?

The “Action type” and “Intervention function” are interesting but these do not map onto the theory of change, as far as I can tell. A better description of these categories and what they mean would be valuable.

*Response*

Both reviewers commented on Table 1, which means that it was not clear enough. In order to understand what kinds of individual and collective action people might take, and therefore to align our expectations in the theory of change with potential reality, we documented previous actions. Table 1 was included simply to give some illustrations through which readers could imagine the situation. We have redrafted it substantially, including columns categorising actions as individual, collective, or individual followed by collective, whether an action was in the home or the neighbourhood, and the type of violence to which it was a response. The core team (Daruwalla, Gram, Osrin) have selected stories purposively to illustrate combinations of these categories: again, to give readers a flavour of the kinds of things that were done. We have retained the column categorising intervention functions in the COM-B model because we think it is educative. We have included more background on all of this in the Methods:

“In order to understand what kinds of individual and collective action people might take, and therefore to align our expectations in the theory of change with potential reality, the action documentation exercise recorded community mobilisation team members’ experiences of the kinds of action that community members had undertaken in the past. It yielded 76 actions, documented as extended data (Osrin, 2019). Adverse effects were also documented (although flare-ups in communities might actually suggest that the program was having an effect).”

“Table 1 provides examples of actions, categorising them as individual, collective, or individual followed by collective, whether an action was in the home or the neighbourhood, and the type of violence to which it was a response. The core team selected stories purposively to illustrate combinations of these categories. Individual action was often backed by the notional and practical security of being a member of a community group and having connections with a supportive organisation, using these connections as action escalated. Apart from help from our own organisation, and from periodic referral for skilled legal help and services for alcohol and drug dependency, the police force was a prominent institutional link. Although our commitment is to preventing domestic violence, both individual and collective actions often responded to incidents in the neighbourhood. Different kinds of violence – and particularly sexual violence in public spaces – coexist and response to each is both a source of confidence and a step toward a belief in collective efficacy. Likewise, groups often undertook collective action to improve their environment and we see this as contributing to a sense of collective efficacy and community building.”

“We also tried to classify behaviour change according to the Capability, Opportunity, and Motivation model (COM-B), a theoretical approach that we will use in subsequent process evaluation. Developed to inform public health programming, the COM-B model specifies nine functions by which an intervention might stimulate change: education, persuasion, incentivisation, coercion, training, restriction, environmental restructuring, modelling, and enablement. The examples in Table 1 point to education (awareness of the problem of violence and potential approaches to addressing it), persuasion (of either perpetrators or community bodies), coercion (primarily of perpetrators, and often through linkages with the police), and modelling (of exemplary successful actions by individuals and groups) as prominent functions.”

*Comment 2*

The theory of change itself in Figure 1 adds considerably to the literature. I am particularly impressed by the assumptions that are articulated. However, I’m concerned that the arrows leading from “Resources” to “Changes” seem to be, in and of themselves, something of a black box. I would like to know (and the IPV field is search for answers to the question of) how community volunteers and groups of women and men work together to change participants? Indeed, how do these volunteers and community members take up their work in such a way that communities change overall? Is it through solidarity and group mutual support? Or is it through personal change that leads to an overall community-level benefit (i.e. the whole is greater than the sum of its parts)? The mechanism of action is not identified, which makes it very challenging to test the underlying assumptions of the theory. One suggestion would be to highlight important Theory (as in, sociological or behavioral theory) either within the figure or the accompanying text.

*Response*

These comments are well taken and we have added material to the Results and Discussion sections. We think that the reviewer’s suggestions are both correct: the intervention develops solidarity and group support, along with collective efficacy and action, and also personal change in terms of capability, opportunity, and motivation to take action.

In Results: “The theory of change narrative is that people involved in our intervention take action to help survivors of violence make informed choices, and to increase community awareness of violence against women and girls and the possibility of change. As a result of these activities, survivors and potential perpetrators understand the nature of violence. Survivors make decisions, potential perpetrators think again, and other people understand both the nature of violence and that action is possible. People stand up against violence against women and girls, individually and collectively, and community members think and act to help survivors. Families and communities stop accepting violence and strengthen community structures that support a conviction that it is intolerable. Institutional support from non-government organisations, the police, medical practitioners, and lawyers is accessible and functional and families and communities free themselves of violence against women and girls.”

In Discussion: “A potential criticism of a theory of change is that the connections between specific activities and outcomes are often insufficiently understood, a challenge that we call the *block and arrow* problem. Given our theory’s provisional nature, we hope to open up the blocks to understand the ways in which their components ‘work’. This is clearly difficult in a complex system with multiple inputs and emergent phenomena, but can be divided into three projects. The first project involves the stuff of process evaluation: to understand what was delivered, how well, what was received, and by whom. An evaluation framework is in place to address these questions both qualitatively and quantitatively in terms of reach, efficacy, adoption, implementation, and maintenance (the RE-AIM framework (Glasgow, Vogt, & Boles, 1999), which we have used before (Shah More et al., 2013)). The second project is to understand whether each of the components of the three boxes describing change happens. We hope to evaluate this quantitatively through an electronic intervention monitoring system and simultaneous qualitative observation and interview: each of the changes listed in the boxes has been taken as an indicator. For example, we will track bystander intervention quantitatively and through case studies. We will enumerate identification of survivors and their subsequent communication with medical, police, and legal services. We will conduct qualitative and quantitative assessments of people’s understanding of violence and the degree to which it ceases to be thought of as a private family matter. We will follow group membership, actions taken by members, and the development of leadership. And we will document community enquiries and referrals as a result of violence against women.”

“The third project is to understand the mechanisms through which program effects are achieved. Like many public health implementors, we have a strong interest in realist evaluation (Pawson & Tilley, 1997). Within an intervention with many moving parts, which activities work well, for whom, and in which contexts? Our aim is to generate candidate context-mechanism-outcome configurations that we can propose as hypotheses, and to test them. The context of program sites will be described after a combination of ethnographic work, participatory learning and action activities, and quantitative data collection on demography, sociocultural indices, and experience of and response to violence against women. We take a method-neutral approach, combining ethnographic interviews and observation, targeted qualitative interviews with staff, group members, and volunteers, and case studies of how the intervention unfolds in selected localities; these augmented with data from our intervention monitoring system that can be used to answer specific questions. Central issues for consideration include the contribution of secondary prevention to primary prevention, the role of collective action (Gram, Daruwalla, & Osrin, 2019), the role of expanded reference groups in norm change, and the role of men in program effectiveness (Chakraborty, Osrin, & Daruwalla, 2018).”

*Comment 3*

The notion of a “ceiling of accountability” is interesting, and I wonder if the authors can discuss how many IPV prevention programs position themselves along these lines. While many programs cite their stated aim is to reduce domestic violence, few projects globally have ever proven that they are able to do this.

*Response*

A ceiling of accountability is recommended by experts in theory of change. We are not aware of other theories from violence prevention programs having used it. It is particularly relevant because of the need to be realistic about what a program could achieve. We do aim to reduce domestic violence, but wider effects are unlikely in the short term.

*Comment 4*

It would be nice to hear a bit more about how sanginis “emerge” (or are chosen by the program to take up this role).

*Response*

We have added to the Results section:

“Group sessions aim to build a political agenda for change that privileges women’s interests and looks to transform gender and social power relations. Our group-work module follows a sequence over three years. The first year emphasises awareness of violence, gender norms, women’s rights and entitlements, and the importance and strength of women’s collectives in addressing violence against women and girls. The second year emphasises action, be it individual or collective. The third year emphasises leadership and group members are encouraged to take on prominent roles in the community. Generally, women who are proactive, articulate, have a social network in their neighbourhoods, and are willing to devote time to the issue are recommended as volunteer activists by groups and invited to become *sanginis*.”

*Comment 5*

As above, the discussion statement around “makes explicit specific testable causal pathways to impact” is not entirely right, since the pathways themselves are poorly fleshed out. While the ‘context’ and ‘outcome’ configurations are indeed mapped out, I believe the ‘mechanism’ linking the two requires additional thinking.

*Response*

This challenge has been articulated by both reviewers. We have added information about our evaluative plans to the Discussion section.

“A potential criticism of a theory of change is that the connections between specific activities and outcomes are often insufficiently understood, a challenge that we call the *block and arrow* problem. Given our theory’s provisional nature, we hope to open up the blocks to understand the ways in which their components ‘work’. This is clearly difficult in a complex system with multiple inputs and emergent phenomena, but can be divided into three projects. The first project involves the stuff of process evaluation: to understand what was delivered, how well, what was received, and by whom. An evaluation framework is in place to address these questions both qualitatively and quantitatively in terms of reach, efficacy, adoption, implementation, and maintenance (the RE-AIM framework (Glasgow et al., 1999), which we have used before The second project is to understand whether each of the components of the three boxes describing change happens. We hope to evaluate this quantitatively through an electronic intervention monitoring system and simultaneous qualitative observation and interview: each of the changes listed in the boxes has been taken as an indicator. For example, we will track bystander intervention quantitatively and through case studies. We will enumerate identification of survivors and their subsequent communication with medical, police, and legal services. We will conduct qualitative and quantitative assessments of people’s understanding of violence and the degree to which it ceases to be thought of as a private family matter. We will follow group membership, actions taken by members, and the development of leadership. And we will document community enquiries and referrals as a result of violence against women.”

“The third project is to understand the mechanisms through which program effects are achieved. Like many public health implementors, we have a strong interest in realist evaluation (Pawson & Tilley, 1997). Within an intervention with many moving parts, which activities work well, for whom, and in which contexts? Our aim is to generate candidate context-mechanism-outcome configurations that we can propose as hypotheses, and to test them. The context of program sites will be described after a combination of ethnographic work, participatory learning and action activities, and quantitative data collection on demography, sociocultural indices, and experience of and response to violence against women. We take a method-neutral approach, combining ethnographic interviews and observation, targeted qualitative interviews with staff, group members, and volunteers, and case studies of how the intervention unfolds in selected localities; these augmented with data from our intervention monitoring system that can be used to answer specific questions. Central issues for consideration include the contribution of secondary prevention to primary prevention, the role of collective efficacy (Gram et al., 2019), the role of expanded reference groups in norm change, and the role of men in program effectiveness (Chakraborty et al., 2018).”

*Comment 6*

The protocol by Christofides et al (2016) would be important to include in the discussion, given its emphasis on a theory of change for primary prevention of IPV in a similar peri-urban setting. This would be particularly valuable in the sections on the three types of theory and the section about how policy change is incorporated into a theory of change and the concomitant impact evaluation.

*Response*

We actually discussed this protocol in a recent team seminar, but had not included protocols in our paper. We have added text in the relevant section.

“A similar approach has been taken in the development of Sonke CHANGE, a cluster randomised controlled trial in periurban Johannesburg, South Africa. The Sonke Gender Justice intervention involves workshops and community action teams working predominantly with men with an emphasis on changing harmful gender norms and prevailing hegemonic masculinity. The program theory of change proposes that community action and advocacy to promote equitable gender norms and non-violent masculine attitudes and practices will lead to enhanced critical consciousness, collective efficacy and action, and better social cohesion and trust. These will lead in turn to self-efficacy to take action, reduced influence of harmful gender norms, and an enabling environment for policy implementation (Christofides et al., 2018).”

*Comment 7*

What methods beyond running a trial are the authors engaged with presently? For example, will they harness path analysis to unpack the mechanisms underlying intervention success? Will they conduct a qualitative process evaluation alongside the trial?

*Response*

Unfortunately, we are still waiting for the trial protocol to be published. It contains a fairly comprehensive discussion of what we intend to do. We are doing a qualitative and quantitative process evaluation as part of the trial, along with the development and testing of context-mechanism-outcome configurations. We have added information on to the Discussion section and it is included in the response to Comment 5.

*Comment 8*

While the contradictions between feminist framing and the needs of trials/projects to be managerial in nature is important, I wonder if it could use additional thinking. In particular, this is an area where Theory (capital T) could be brought forward more intentionally, since the authors are clearly drawing from multiple approaches and epistemologies.

*Response*

In light of this and other comments, we have added a discussion of theory to the Results.

“Three areas of disciplinary theory meet in the ideas that underlie our program theory: social norms theory (already prominent in prevention of violence against women), network theory (prominent in political science, economics, and latterly in public health), and behaviour change theory (prominent in behavioural psychology and public health).”

“Our ideas about norms are informed by feminist theory (Heise, 1998), which also underlies our organizational approach to working with survivors of violence. Beyond the need to work with families, we aim to ensure that no further harm is done to women, who remain the focus and whose views and making of meaning are prioritized. We believe that violence against women deserves attention as a gendered phenomenon beyond family violence (Lawson, 2012), and that gender needs to be in the foreground (Dobash & Dobash, 1979; Gelles, 1985). Our attempts to achieve gender transformative change are particularly indebted to theory around asymmetric distribution of power between genders (Rodrígues-Menés & Safranoff, 2012), and hegemonic patriarchy (R. Connell, 1987; R. W. Connell, 1995).”

“Our thinking on potential mechanisms has been influenced by social norms theory, and particularly by discussions of the need to transform gender norms as a route to addressing violence against women (Alexander-Scott, Bell, & Holden, 2016). This is particularly relevant because we are concerned about the forms of violence that constitute gender-based household maltreatment: emotional and economic violence, control and neglect. Our previous work suggested that we might focus on three ideas about norm change (Daruwalla et al., 2017). The first is to make use of the mismatch between descriptive and injunctive norms. A descriptive norm describes beliefs about what other people do (Bicchieri, 2006; Cialdini, Kallgren, & Reno, 1991; Muldoon, Lisciandra, Bicchieri, Hartmann, & Sprenger, 2014), while an injunctive norm describes beliefs about what other people think one ought to do (Bicchieri, 2006; Cialdini et al., 1991). Descriptive norms intolerant of violence are likely to be magnets for behaviour and if we are able to show examples of non-violent interaction we might be able to influence people’s perception of what is actually prevalent in the community. This is helped by our second idea, which is that injunctive norms disapprove (at least in principle) of violence. How to spread awareness of this turns on the reference group - the people surrounding an individual from whom she takes her cue (Levy Paluck, Ball, Poynton, & Sieloff, 2010) - and our third idea is to expand the currently limited reference groups around individuals so that they can process a larger pool of opinion on norms intolerant of violence.”

“When we ‘expand a reference group’, we are basically saying that individuals meet and engage with more people, and this evokes ideas from network or social capital theory (Putnam, 1995). Another way of seeing the new connections between people that develop in community groups is as weak ties or bridging social capital, in contradistinction to the (hierarchical) strong ties or bonding social capital that the family circle typifies (Granovetter, 2005; Patulny & Svendsen, 2007). Added to this is the idea of linking social capital between, for example, community members and institutions that might help them to address violence against women (Szreter & Woolcock, 2004), a process explicit in our theory of change. So, although community mobilisation is a way to address violence against women, it can be seen equally as a community building or social capital endeavour (see (Szreter & Woolcock, 2004) for an anatomisation of the debates around social capital and public health).”

“We use the Capability, Opportunity, and Motivation model (COM-B) to think about individual behaviour change, particularly in terms of the functions of interventions that it summarises (Michie, van Stralen, & West, 2011). The program aims to increase the capability of participants to understand violence against women, identify survivors, and take supportive action, with knowledge of law and rights, decision-making and negotiation skills, self-efficacy, and collective efficacy. This it does predominantly through education, training, enablement, and modelling (first by program staff and then by community members). It presents opportunities to engage with others, learn, develop confidence and leadership, and connect with supportive non-government and government organisations; again predominantly through education, training, enablement, and modelling. Motivation comes from the development of belief in preventing violence against women and children, increased by education, persuasion, enablement, training, and modelling. From the point of view of potential perpetrators of violence, the primary functions are persuasion, coercion, and education, accompanied (we hope) by awareness and change in attitude brought about by education and modelling and reduced opportunity for violent behaviour.”

**References**

Alexander-Scott, M., Bell, E., & Holden, J. (2016). *DFID Guidance Note: shifting social norms to tackle violence against women and girls (VAWG)*. London.

Bicchieri, C. (2006). *The grammar of society: the nature and dynamics of social norms*. Cambridge: Cambridge University Press.

Chakraborty, P., Osrin, D., & Daruwalla, N. (2018). “We Learn How to Become Good Men”: Working with Male Allies to Prevent Violence against Women and Girls in Urban Informal Settlements in Mumbai, India. *Men & Masculinities*, 1-23. doi: 10.1177/1097184X18806544

Christofides, N. J., Hatcher, A. M., Pino, A., Rebombo, D., McBride, R. S., Anderson, A., & Peacock, D. (2018). A cluster randomised controlled trial to determine the effect of community mobilisation and advocacy on men's use of violence in periurban South Africa: study protocol. *BMJ Open, 8*(3), e017579. doi: 10.1136/bmjopen-2017-017579

Cialdini, R., Kallgren, C., & Reno, R. (1991). A focus theory of normative conduct: a theoretical refinement and reevaluation of the role of norms in human behavior. *Adv Experimental Soc Psychol, 24*, 201-234

Connell, R. (1987). *Gender and power: society, the person and sexual politics*. Stanford CA: Stanford University Press.

Connell, R. W. (1995). *Masculinities*. Berkeley & Los Angeles CA: University of California Press.

Daruwalla, N., Hate, K., Pinto, P., Ambavkar, G., Kakad, B., & Osrin, D. (2017). You can't burn the house down because of one bedbug: a qualitative study of changing gender norms in the prevention of violence against women and girls in an urban informal settlement in India. *Wellcome Open Res, 2*, 48. doi: 10.12688/wellcomeopenres.11805.2

Dobash, R. E., & Dobash, R. P. (1979). *Violence against wives: a case against the patriarchy*. New York: Free Press.

Gelles, R. J. (1985). Family violence. *Ann Rev Sociol, 11*, 347-367

Glasgow, R. E., Vogt, T. M., & Boles, S. M. (1999). Evaluating the public health impact of health promotion interventions: the RE-AIM framework. *Am J Public Health, 89*, 1322-1327

Gram, L., Daruwalla, N., & Osrin, D. (2019). Understanding participation dilemmas in community mobilisation: can collective action theory help? *J Epidemiol Community Health, 73*(1), 90-96. doi: 10.1136/jech-2018-211045

Granovetter, M. (2005). The impact of social structure on economic outcomes. *J Econ Perspectives, 19*, 33-50

Heise, L. (1998). Violence against women: an integrated ecological framework. *Violence Against Women, 4*, 262-290

Lawson, J. (2012). Sociological theories of intimate partner violence. *J Hum Behav Soc Environment, 22*, 572-590

Levy Paluck, E., Ball, L., Poynton, C., & Sieloff, S. (2010). *Social norms marketing aimed at gender based violence: a literature review and critical assessment*. New York.

Michie, S., van Stralen, M. M., & West, R. (2011). The behaviour change wheel: a new method for characterising and designing behaviour change interventions. *Implement Sci, 6*(42). doi: 10.1186/1748-5908-6-42

Muldoon, R., Lisciandra, C., Bicchieri, C., Hartmann, S., & Sprenger, J. (2014). On the emergence of descriptive norms. *Politics Philosophy Econ, 13*, 3-22

Patulny, R. V., & Svendsen, G. L. H. (2007). Exploring the social capital grid: bonding, bridging, qualitative, quantitative. *Int J Sociol Soc Policy, 27*, 32-51

Pawson, R., & Tilley, N. (1997). *Realistic evaluation*. London: SAGE.

Putnam, R. (1995). Bowling alone: America's declining social capital. *J Democracy, 6.1*, 65-78

Rodrígues-Menés, J., & Safranoff, A. (2012). Violence against women in intimate relations: a contrast of five theories. *Eur J Criminol, 9*, 584-602

Shah More, N., Das, S., Bapat, U., Rajguru, M., Alcock, G., Joshi, W., . . . Osrin, D. (2013). Community resource centres to improve the health of women and children in Mumbai slums: study protocol for a cluster randomized controlled trial. *Trials, 14*, 132. doi: 10.1186/1745-6215-14-132

Szreter, S., & Woolcock, M. (2004). Health by association? Social capital, social theory, and the political economy of public health. *Int J Epidemiol, 33*, 650-667
